# A Microfluidic DNA Library Preparation Platform for Next-Generation Sequencing

**DOI:** 10.1371/journal.pone.0068988

**Published:** 2013-07-22

**Authors:** Hanyoup Kim, Mais J. Jebrail, Anupama Sinha, Zachary W. Bent, Owen D. Solberg, Kelly P. Williams, Stanley A. Langevin, Ronald F. Renzi, James L. Van De Vreugde, Robert J. Meagher, Joseph S. Schoeniger, Todd W. Lane, Steven S. Branda, Michael S. Bartsch, Kamlesh D. Patel

**Affiliations:** 1 Department of Biotechnology and Bioengineering, Sandia National Laboratories, Livermore, California, United States of America; 2 Department of Systems Biology, Sandia National Laboratories, Livermore, California, United States of America; 3 Advanced Systems Engineering and Deployment, Sandia National Laboratories, Livermore, California, United States of America; Natural History Museum of Denmark, University of Copenhagen, Denmark

## Abstract

Next-generation sequencing (NGS) is emerging as a powerful tool for elucidating genetic information for a wide range of applications. Unfortunately, the surging popularity of NGS has not yet been accompanied by an improvement in automated techniques for preparing formatted sequencing libraries. To address this challenge, we have developed a prototype microfluidic system for preparing sequencer-ready DNA libraries for analysis by Illumina sequencing. Our system combines droplet-based digital microfluidic (DMF) sample handling with peripheral modules to create a fully-integrated, sample-in library-out platform. In this report, we use our automated system to prepare NGS libraries from samples of human and bacterial genomic DNA. *E. coli* libraries prepared on-device from 5 ng of total DNA yielded excellent sequence coverage over the entire bacterial genome, with >99% alignment to the reference genome, even genome coverage, and good quality scores. Furthermore, we produced a *de novo* assembly on a previously unsequenced multi-drug resistant *Klebsiella pneumoniae* strain BAA-2146 (KpnNDM). The new method described here is fast, robust, scalable, and automated. Our device for library preparation will assist in the integration of NGS technology into a wide variety of laboratories, including small research laboratories and clinical laboratories.

## Introduction

Next-Generation Sequencing (NGS) technology [1] has been widely adopted for applications ranging from microbial community profiling [2], [3] to screening for genetic abnormalities associated with human disease [4], [5] to pathogen discovery and characterization [6], [7]. NGS offers massively parallel data collection that enables genome, transcriptome, or metagenome sequencing to be performed with a single instrument at a cost of a few thousand dollars *per* run [8]. Moreover, a new generation of “personal” instruments such as MiSeq (Illumina), GS Junior (454 Life Sciences), and Ion Torrent (Life Technologies) have made NGS increasingly accessible to small and resource-limited laboratories [9], including clinical laboratories.

Although NGS technology itself has already transformed genomic research, the preparation of properly formatted sequencing libraries from DNA or RNA samples is a lengthy, multi-step process. While there is great interest in developing streamlined techniques for preparing sequencer-ready libraries [1], few automated solutions have been described, and most library preparation methods used today are manual and tedious. Robotic platforms can be configured for high-throughput library preparation, but are not cost-effective for smaller laboratories that have inconsistent demand, limited resources, or need for greater flexibility in customizing the workflow for a wide variety of samples types. Furthermore, pipette-based techniques – whether robotic or manual – are difficult to scale down to volumes less than a few microliters, which makes efficient preparation of libraries from small or limited samples (as commonly encountered in clinical applications) challenging.

We report here a rapid, automated solution for preparing Illumina sequencer-ready libraries from only a few nanograms of genomic DNA. This method is enabled by distinct capillary-based modules integrated through digital microfluidics (DMF) – a fluid-handling technique in which discrete droplets of samples and reagents are actuated on a planar hydrophobic surface by applying a series of electrical potentials to an array of electrodes [10]–[12]. DMF excels at discrete “pipette-like” fluidic manipulations including reagent dispensing, mixing and splitting of aliquots at the low-microliter or sub-microliter scale. Protocols for sequencing library preparation typically consist of a series of “unit operations” including physical or enzymatic transformations, cleanup, temperature-controlled amplification, and size selection steps [13]–[15]. Although many of these individual unit operations [16]–[18] might conceivably be performed within the context of a DMF device, integrating all of these functionalities with disparate requirements onto a single platform is challenging.

We recently described a robust, standardized capillary-DMF interface to interconvert liquid samples between the discrete droplet DMF format and a continuous-flow format for straightforward, programmable sample preparation steps including dispensing, fractionation and separation [19], [20]. Building on our initial efforts, we introduce here a fully integrated and automated microfluidic system, in which the DMF platform performs basic reagent metering and mixing steps, but also serves as a “hub” or “fluidic router” to integrate the operation of off-DMF modules that are individually optimized to perform each of the disparate steps in sequencing library preparation.

**Figure 1:**
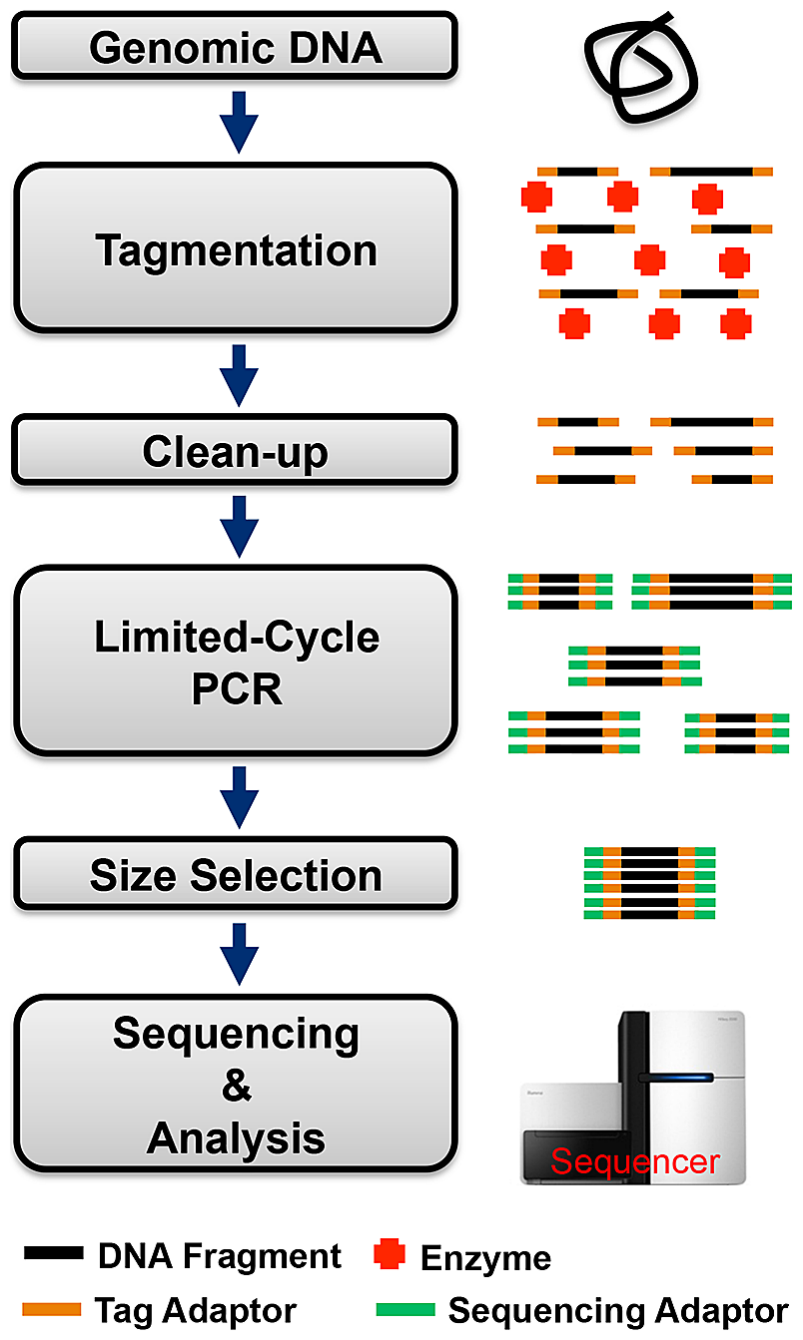
Nextera® protocol for preparing DNA libraries compatible with Illumina sequencers. Key steps include: gDNA tagmentation, clean-up, limited-cycle PCR, and selection of size-specific DNA library for sequencing and analysis.

To test the performance of our new method, human and bacterial genomic DNA (gDNA) libraries were prepared using the Nextera sample preparation protocol [21] on our platform, and libraries were sequenced in-house using the Illumina MiSeq system. The Nextera sample preparation protocol is itself streamlined relative to traditional sample preparation techniques [22] that involve mechanical fragmentation of DNA followed by end repair, dA tailing, and adapter ligation. In the Nextera workflow, shown schematically in Figure 1, gDNA is first “tagmented” in a transposase-mediated reaction that simultaneously fragments and tags DNA with adapters. The adapter-tagged DNA fragment library is then purified to remove unwanted constituents from the tagmentation reaction. Sequencing adapters are added to the fragment library by limited-cycle PCR, and finally the DNA library is size-selected for sequencing and analysis. Our DMF hub with peripheral modules automates this entire workflow into a single, hands-free protocol. The differentiating strength of this platform is that it can be easily reconfigured and reprogrammed to accommodate a wide range of library preparation techniques for diverse applications. Further, the platform is easily scalable with respect to the volume and number of samples, allowing efficient processing of precious samples.

## Experiments

### Reagents and Materials

Fluorinert FC-40, Pluronic F127, ethanol and 1X Tris-EDTA (TE) (pH 8.0) buffer were purchased from Sigma Chemical (St. Louis, MO). Parylene C dimer was purchased from Specialty Coating Systems (Indianapolis, IN), Teflon-AF from DuPont (Wilmington, DE), Nextera DNA Sample Preparation Kit from Illumina (San Diego, CA) and SPRI (Solid Phase Reversible Immobilization) magnetic beads from Beckman Coulter (Danvers, MA). Human genomic DNA isolated from peripheral blood mononuclear cells, *Escherichia coli* MG1655, and *Klebsiella pneumoniae* strain BAA-2146 genomic DNA were purchased from American Type Culture Collection (ATCC) (Manassas, VA).

Working solutions of all DNA were prepared in TE buffer with 0.1% Pluronic F127 (w/v) [23], [24]. For DNA library preparation on the DMF hub, Nextera mixture (2 µL High Molecular Weight, 0.5 µL enzyme mix), Nextera PCR mixture (12.5 µL 2X PCR buffer, 0.5 µL primer cocktail, 0.5 µL PCR enzyme, 0.5 µL 50X Adapter 2) and elution buffer (water with 0.1% Pluronics F127 w/v) were used. HPLC-grade organic solvents and nuclease-free water were used in all experiments.

### Bacterial Growth Conditions and DNA Extraction

*E. coli* MG1655 was plated on Luria Broth (LB) agar plates. An isolated colony was inoculated into 5 ml of LB broth and grown overnight at 37°C with shaking. A 50 µl aliquot of the overnight culture was used to inoculate 5 ml of fresh LB broth and the bacteria were incubated as before for 2 hours, at which point exponential growth had been established. DNA was extracted from 1 ml of bacterial culture using the DNeasy Blood and Tissue Kit according to the manufacturer’s instructions (Qiagen). The extracted DNA was quantified by Qubit (Life Technologies, Grand Island, NY) prior to library generation.

### DMF Device Fabrication and Assembly

**Figure 2:**
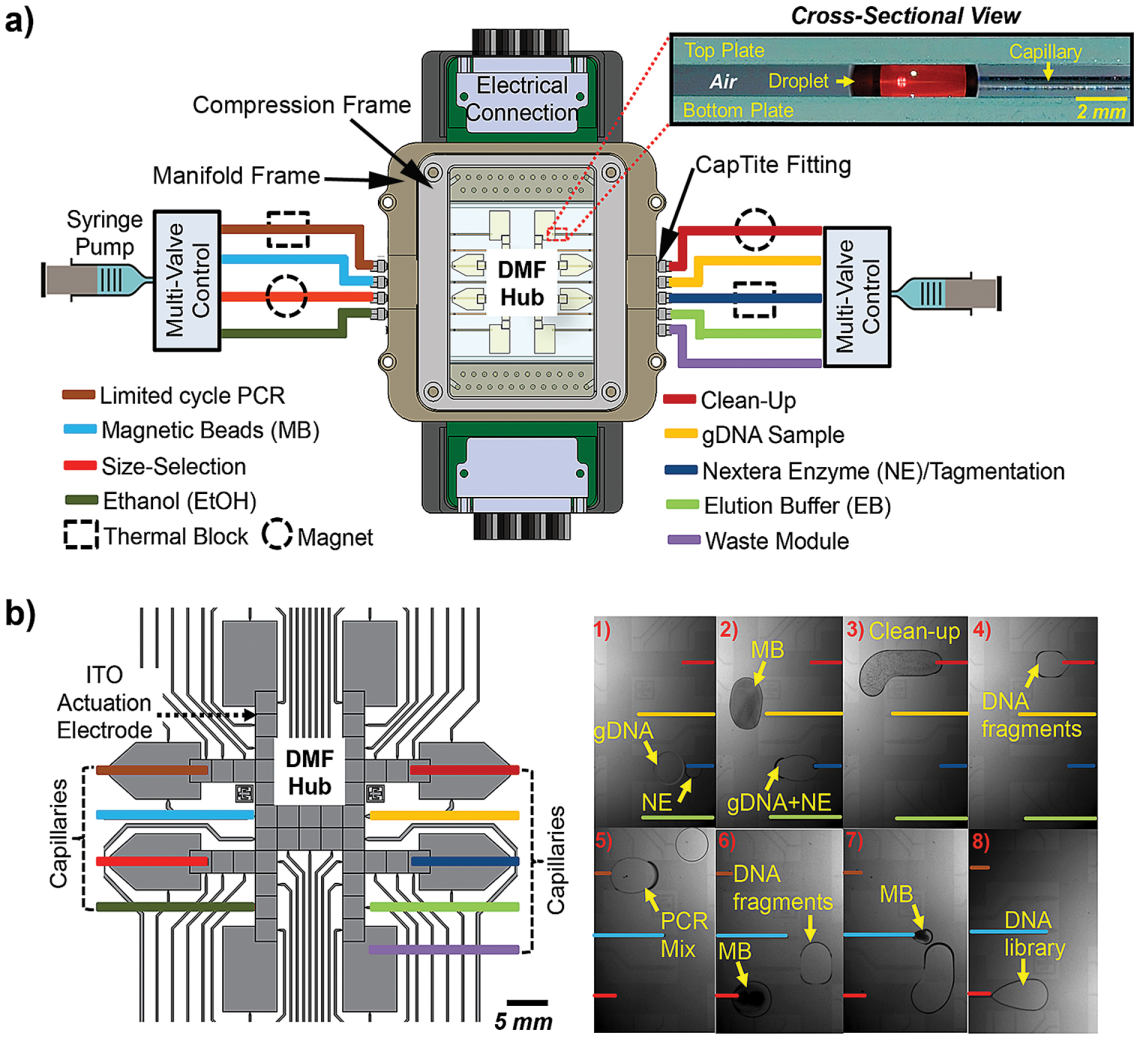
Integrated microfluidic system for preparing DNA libraries for sequencing. a) Top view of an integrated system and side view of the DMF-capillary interface, which features a central DMF hub for integrating multiple reagent and sample preparation modules (depicted in different colors), magnets and thermal blocks coupled to module tubing for sample preparation, and multi-valve syringe pumps for liquid handling. b) Schematic (left) of a DMF device showing the arrangement of indium tin oxide (ITO) actuation electrodes used to route samples and reagents to and from the capillaries, which are responsible for transferring liquids to their respective modules. Sequence of frames (right) from a movie illustratfing the stages in preparing a sequencer-ready DNA library using the microfluidic method (note that off-DMF processing is not shown in the perspective of the frames): (1) Mixing of gDNA (1.8 µL) and Nextera Enzyme (NE, 0.6 µL) droplets; (2,3) post-tagmentation droplet (gDNA+NE reaction products) merged with magnetic bead (MB) droplet (4.5 µL) and actuated to clean-up module; (4,5) post-clean-up droplet (2.2 µL) of purified DNA fragments actuated to PCR Mix droplet (2.8 µL) for limited-cycle PCR; (6,7) post-PCR DNA fragment droplet mixed with different volumes (2.2 and 0.45 µL) of magnetic beads for size-selection; and (8) DNA library droplet (3 µL).

DMF devices were fabricated in the Sandia National Laboratories Applied Biosystems Laboratory cleanroom facility, using a transparent photomask printed at Photo Sciences (Torrance, CA). Glass devices bearing patterned indium tin oxide (ITO) electrodes were formed by photolithography and etching as described previously [19], [20], [25], and were coated with 4 µm of Parylene-C and 50 nm of Teflon-AF. Parylene-C was applied using a vapor deposition instrument (Specialty Coating Systems, Indianapolis, IN), and Teflon-AF was spin-coated (1% wt/wt in Fluorinert FC-40, 2000 rpm, 60 s) followed by post-baking on a hot-plate (160 °C, 10 min). Polyimide tape (DuPont, Hayward, CA) was placed on the electrode contact pads prior to parlyene coating and was removed after coating to enable electrical contact. In addition to patterned devices, unpatterned indium tin oxide (ITO) coated glass substrates (Part # CG-41IN-S207; Delta Technologies Ltd, Stillwater, MN) were coated with Teflon-AF (50 nm, as above) to serve as device top plates. The device design (Figure 2b) features an array of forty-eight actuation electrodes with inter-electrode gaps of 20 µm, forty-four of which are rectangular (i.e., forty ∼ 2.5×2.5 mm and four ∼ 6.3×9.5 mm each) and four pentagon-shaped (∼ 4.5×7.5×6.3 mm each).

The DMF hub used here is identical to those described elsewhere [19], [20]. DMF hubs with recesses that maintain a 185 or 400 µm gap spacing between the top and bottom plates were used to accommodate different droplet volumes. In brief, DMF plates were fixtured in a custom-engineered polymer manifold frame using metal compression frames to hold the unpatterned ITO–glass top plate and the patterned electrode plate into precisely spaced recesses in the manifold frame. Fourteen side ports provide access to the DMF device by enabling the insertion and registration of individual capillaries into the space between the two DMF plates. Electrical contact to the DMF plates was made by spring-loaded pogo pins partially recessed in the manifold frame.

### DMF Hub–Module Interface

To form the DMF Hub–module interface, 3.5–4.0 cm long, fused-silica capillaries (Polymicro, Phoenix, AZ) coated in-house with Teflon-AF were inserted through the side ports and into the air space between the DMF plates. For DMF Hubs with 185 µm spacing, 160 µm outer diameter (OD) and 100 µm inner diameter (ID) capillaries (Part # TSU100170) were inserted and positioned ∼ 250 µm away from the edge of the receiving 2.5×2.5 mm DMF actuation electrodes. The distal ends of the capillaries were connected to larger volume polycarbonate tubing modules with 750/562 µm OD/ID (Part # CT562-750-5; Paradigm optics, Vancouver, WA), which served as sample processing and reagent reservoirs (Figure 2a). Short coupling tubes with 500/167 µm OD/ID (Part # CT250-500-5) assisted in bridging the difference in capillary-to-tubing diameter. The multidiameter tube assembly was glued with optical cement (Norland, Cranbury, NJ) to fluidically seal the junction. For DMF Hubs with 400 µm spacing, 360/100 µm OD/ID capillaries (Part # TSU100375) were directly connected (by insertion) to 375/750 µm OD/ID tubing (Part # CT375-750-5) modules, and swaged into the side ports with CapTite fittings (Sandia National Labs, Livermore, CA). Lastly, the distal ends of tubing modules were connected to an 8-port valve manifold at the head of a syringe pump (Part # 59943-01; Hamilton, Reno, NV) to provide positive displacement pumping and enable the dispensing, aspiration, and transfer of microliter-sized fluid droplets and boluses between the central DMF device and external modules.

### System Operation

DMF droplet actuation was executed by a custom computer-controlled electronic interface, either by manual keystrokes or script-based preprogrammed sequences. To actuate droplets, driving potentials (80–90 V_RMS_) were generated by amplifying (Digi-Key Corporation, Thief River Falls, MN) the output of a function generator (Trek, Medina, NY) operating at 15 kHz. As described in greater detail elsewhere [19], droplets sandwiched between the two plates were actuated by applying driving potentials sequentially between electrodes patterned on the bottom plate and the top electrode (ground).

For this demonstration, DNA sample and reagents were manually loaded into capillaries pre-primed with water to provide an incompressible coupling to the syringe pump for precise sub-microliter metering. A 1-2 µL air gap separated the reagent/reaction bolus from the pumping water. Fluid boluses were moved to and from the DMF device using syringe pumps (at a flow rate of 10 µL/min) controlled by custom software. An MVX10 microscope (Olympus, Center Valley, PA) with a high speed QIClick digital camera (Qimaging, Surrey, Canada) with a field of view of ∼ 35×26 mm2 was used to observe droplet manipulation on the DMF device. A commercial software package (Streampix5, Norpix, Montreal, Canada) was used to capture video frames to PC.

For DNA tagmentation and limited-cycle PCR, sample tubing was routed through trenches (∼ 850 µm deep×800 µm wide) in custom-machined aluminum block with thermoelectric temperature control provided by an in-house closed-loop temperature controller and benchtop PCR machine (OpenPCR, Saratoga, CA), respectively. For DNA clean-up and size-selection using magnetic beads, a 3 mm diameter×3 mm thick neodymium magnet (K&J Magnetics, Jamison, PA) was manually positioned below and in contact with the clean-up and size-selection modules.

### DNA Library Preparation on the Platform

For DNA Library preparation, the Nextera DNA Sample Preparation Kit (Illumina, San Diego, CA) was used following manufacturer’s instructions and implemented on the DMF Hub. For human genomic DNA samples prepared on a 185 µm-gap DMF Hub, a 1.8 µL droplet containing DNA sample (5 ng/µL) was dispensed onto the DMF platform from a capillary, actuated by DMF and merged with a 0.6 µL droplet of Nextera enzyme. Active mixing (30 s at room temperature) was achieved by continuously aspirating and dispensing the mixture in and out of the capillary. The mixture was then aspirated in the tagmentation module and incubated for 5 min at 55°C.

After tagmentation, the fragmented DNA mixture was dispensed onto the DMF device, merged with a 4.5 µL droplet of magnetic beads, and aspirated into the clean-up module. The DNA/bead mixture bolus was positioned over an external permanent magnet such that beads aggregate along the inner surface of the tube in response to the magnetic field. With the beads immobilized, the supernatant was then returned to the DMF device, actuated to the waste capillary, and aspirated from the device by vacuum to waste. Next, a 3 µL droplet of ethanol (washing solvent) was dispensed onto the DMF device, aspirated into the cleanup module, and moved back-and-forth (30 s) across the immobilized bead pellet to remove trace contaminants. The washing solvent was then dispensed back onto the DMF device, aspirated into the waste module, and aspirated off the device. The washing procedure was repeated two times.

After cleanup, a 2.2 µL droplet of elution buffer was dispensed onto the DMF device, aspirated into the clean-up module, the magnet removed, and beads resuspended in elution buffer (30 s, room temperature). The magnet was then applied again to immobilize the beads, and the DNA-containing elution buffer droplet was dispensed back onto the DMF device where it was mixed (30 s, room temperature) with a 2.8 µL droplet of PCR mixture and actuated to the inlet of the limited-cycle PCR module for addition of sequencing adapters and amplification. 2.8 µL of the droplet mixture was aspirated into the PCR module (the remaining 2.2 µL was actuated to waste) and cycled under the following conditions: 3 min at 72°C and 30 s at 95°C, followed by 10 cycles of 10 s at 95°C, 30 s at 62°C, and 3 min at 72°C.

After limited-cycle PCR, the PCR reaction mixture is dispensed back onto the DMF device for size-selection. A two-stage size selection magnetic bead based cleanup was performed using two different volume ratios of SPRI beads to DNA [26], [27]. First, for removal of small DNA (<200 bp), the reaction mixture was merged with a 2.2 µL droplet of magnetic beads and aspirated into the size-selection module where beads were immobilized by the magnet, and the supernatant was dispensed back onto DMF device. Second, for removal of large DNA (>400 bp), the supernatant was merged with a 0.45 µL volume of magnetic beads and aspirated into the size-selection module where it was washed with ethanol as described above.

Finally, a droplet of elution buffer (3 µL) was dispensed onto the DMF device and actuated to the size-selection module where the DNA library was eluted into the mobile phase and dispensed back onto the DMF device for analysis and sequencing off-chip. For E. coli and K. pneumoniae genomic DNA samples prepared on a 400 µm-gap DMF Hub, the process was identical except: (1) 2X the volume of sample and reagents were used, (2) 9 cycles of PCR were used, and (3) a larger volume (5 µL) of elution buffer was used. Cross-contamination is prevented between different library runs by rinsing the system with a 10% (v/v) solution of bleach and then deionized water.

### Sequencing

After preparation, libraries were quantified for sequencing using the Kapa library quantification kit (KAPA Biosystems, Woburn, MA). The final size of the libraries was determined by Bioanalyzer (Agilent). Sequencing of both *E. coli* and *K. pneumoniae* libraries was performed in-house using individual 300-cycle (paired-end 150 bp) MiSeq kits (Illumina) with a loading concentration of 8 pM. Raw reads have been deposited at National Center for Biotechnology Information (NCBI) Sequence Read Archive under accession SAMN02152539.

### Bioinformatic Analysis

An in-house perl script was used to process raw reads, trimming terminal primer sequences and 3′ and 5′ low quality tails by the algorithm employed in BWA [28], [29], and rejecting reads when fewer than 30 bp remained. Reads generated from *E. coli* libraries were aligned to the reference genome (NC_000913.2) with bowtie2 [29]. De novo assembly was performed using Velvet v1.2.03 [30] with a contig size cutoff of 200 bp and the above expected coverage and molecule length statistics, selecting the best-performing k value after testing with 25, 27 and 29. Library insert size was estimated from bowtie2 mate pair alignments to the *E. coli* genome or to the initial assembly of the KpnNDM genome. Per nucleotide depth was tabulated with the samtools mpileup command. For visualization, read depth was averaged in each of 1000 evenly-spaced, non-overlapping windows across the *E. coli* genome. The Whole Genome Shotgun project for *K. pneumoniae* has been deposited at NCBI Sequence Read Archive under accession APNN00000000.1.

## Results and Discussion

### Development of Integrated Microfluidic System

As shown in Figure 2a, our microfluidic platform comprises a central digital microfluidic (DMF) hub that is interfaced *via* in-plane capillaries to peripheral continuous-flow based sample processing modules that perform DNA “tagmentation”, limited-cycle PCR enrichment, and magnetic bead-based clean-up and size-selection. Two syringe pumps with multiport valves are used as pressure/vacuum sources to deliver and aspirate reagents to the DMF hub and peripheral modules from pre-loaded capillaries (the pumping fluid in the syringes and valves itself is isolated from the reagents bolus by air “spacers”).

The components of the new integrated system were evaluated and optimized to address two concerns: (1) Overcoming nonspecific adsorption of biological molecules to hydrophobic surfaces of the DMF (biofouling), which can impede droplet actuation; and (2) Obtaining reliable coupling of sample processing modules to external components. Regarding biofouling, we found that all sample and reagent droplets could be robustly actuated on DMF surfaces, except for the Nextera enzyme mixture required for the tagmentation process. This was not surprising since there is a high enzyme concentration in tagmentation reagents mixture. In general, high concentration of proteins have shown to be problematic with Teflon-coated DMF surfaces because they tend to adsorb to device surface, resulting in droplet sticking and irreproducible droplet actuation [23], [31]. To alleviate this problem, the nonionic surfactant Pluronic F127 was added at 0.1% (w/v) to the Nextera enzyme solution. Pluronic has previously been shown to reduce surface biofouling in DMF applications, particularly for protein solutions, prolonging device lifetime and inflicting no adverse effects on assays [23], [32]. To confirm that the Pluronic additive did not negatively impact the activity of the Nextera enzyme, we implemented a tagmentation reaction with and without Pluronic added to the Nextera enzyme solution. As shown in Figure S1, the presence of Pluronic does not adversely affect tagmentation efficiency, as the size distribution of reaction products is essentially identical to that observed when Pluronic is not added to the reaction.

A critical consideration for functioning of the thermal modules for tagmentation and limited-cycle PCR was achieving reliable coupling and temperature match between the modules and external heating and cooling elements. This was achieved by fixing the module (750 µm o.d. tubing) in a groove (∼ 850×800 µm, D×W) machined into a temperature-controlled block. We found that if the module and block were only in contact at the surface of the block, there was a significant temperature difference between the temperature of the block and the temperature within the tubing. However, by recessing the module tubing within a groove ∼100 µm below the outer surface of the block, a more reproducible temperature match (<5°C difference) was achieved (see Figure S2), allowing us to reliably perform temperature-dependent reactions in the module. We note that temperature control for droplets on DMF has been demonstrated for systems using oil as a matrix [33]–[35]. Oil-immersed systems have drawbacks, however, including the potential for unwanted liquid–liquid extraction of analytes into the surrounding oil [36], incompatibility with oil-miscible liquids (*e.g.*, organic solvents) and difficulty with extracting product droplet from oil matrix for off-chip analysis [37]. Our platform uses ambient air as the DMF matrix, which combats the above drawbacks, but the air matrix does leave low-volume reagents susceptible to evaporation, especially at high-temperatures. Performing temperature-controlled incubations in tube-based peripheral modules eliminates this concern.

A further requirement for our platform was to implement magnetic bead-based processes (clean-up and size-selection) in peripheral modules. Magnetic bead-based applications including sample preparation [33], [34], immunoassays [33], [38]–[40] and chemical synthesis [41] have previously been executed by actuating particle-laden droplets directly on DMF devices, including key steps such as bead immobilization, separation of supernatant from beads and resuspension of beads. However, the performance and repeated use of magnetic bead-based steps on DMF suffer from several limitations. The gentle droplet actuation forces often result in incomplete separation of supernatant solution from immobilized magnetic bead pellet due to solvent surface tension, which reduces the efficiency of the wash and elution steps. In addition, prolonged immobilization of the bead pellet on the DMF surface can erode the thin Teflon coating, hindering subsequent droplet actuation and promoting biofouling. Finally, bead resuspension and mixing by DMF actuation forces alone can be slow and inconsistent resulting in heterogeneous distribution of beads in droplet solution, although varying magnetic field strengths have been shown to assist in bead resuspension [38].

**Figure 3:**
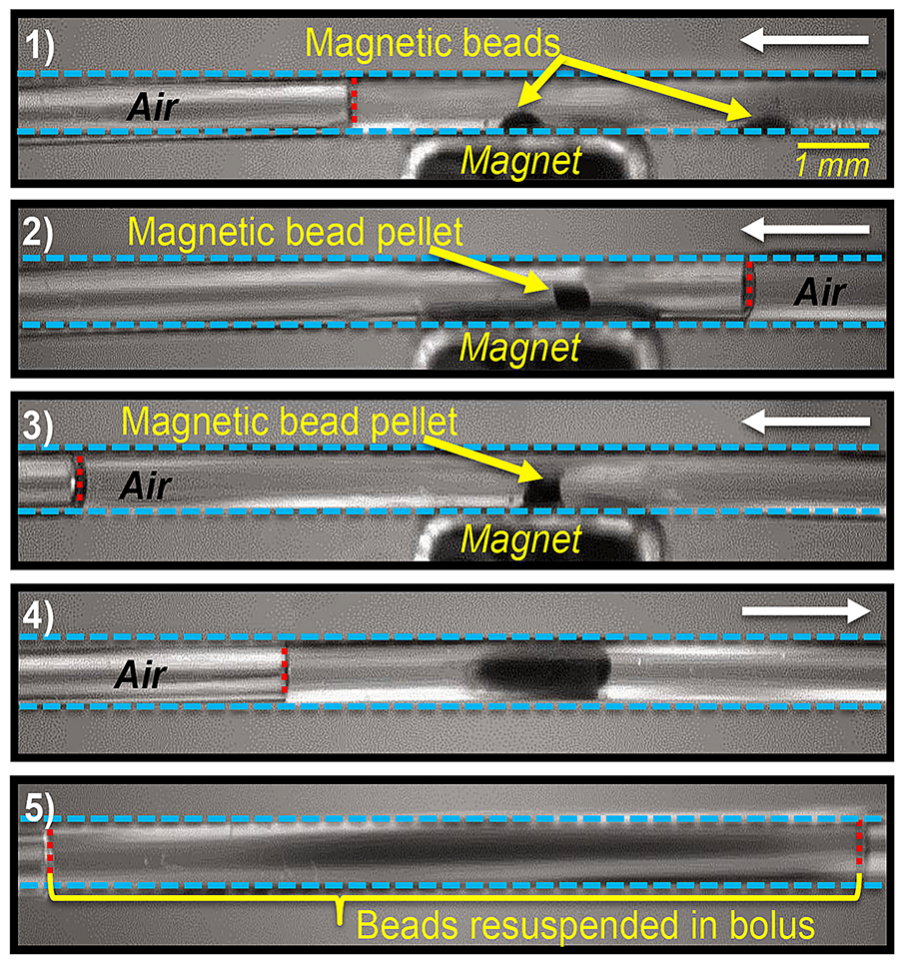
Modular format for implementing magnetic bead-based steps. Series of images from a movie (side-view) depicting key magnetic bead-based steps in an off-DMF module. Blue- and red-dashed lines show the walls of module and air/bolus interface, respectively. (1, 2) An external magnet immobilizes beads onto the tube surface. (3) Positive pressure-driven flow separates supernatant away from the bead pellet. (4, 5) Removing the magnet and shuttling a bolus of solution over the pellet resuspends immobilized beads. Arrows indicate the direction of bolus movement.

Here, we demonstrate a new strategy, in which magnetic bead pulldown, wash, elution, and resuspension steps are implemented in removable or replaceable peripheral modules. In this approach, as shown in Figure 3, a droplet containing magnetic beads is actuated by the DMF to the inlet of the module (not visible in the perspective of Figure 3). Pulling with the syringe pump, the small fluidic bolus with air gaps on either side is aspirated into the module. Next, using an external magnet, the beads are focused into a pellet and immobilized on the inner walls of the module surface (frames 1,2), such that the pellet remains behind when supernatant is displaced away. After removal of supernatant (frame 3), the bead pellet can be subsequently washed, DNA can be extracted from the beads, or beads can be resuspended (frames 4,5) after removing the external magnet, all by driving a bolus of solution over the pellet and repeatedly shuttling the bolus backwards and forwards. Although implementation of this type of configuration is more complex than the conventional DMF approach, the new approach offers several substantial improvements. The most important advantage is that the positive pressure-driven flow (generated by a syringe pump) enables near-complete removal of the supernatant from the bead pellet (Figure 3, frame 3), reducing assay errors and carry-over contamination. Furthermore, shuttling bolus of fluid back-and-forth over the bead pellet (Figure 2b, frame 3) induces a chaotic mixing effect that improves mass transfer in wash and elution steps, and enhances resuspension of beads. Finally, the bead pellet is concentrated outside of the device in a simple disposable capillary tubing, without damaging or pitting the reusable DMF platform itself.

### DNA Library Preparation for Next Generation Sequencing

Preparation of DNA libraries for NGS using the Nextera protocol requires six different reagents (DNA sample, Nextera enzyme, PCR mixture, magnetic bead solution, wash solution, and elution buffer) employed in four distinct processes (tagmentation reaction, clean-up, limited cycle PCR, and size-selection), as shown schematically in Figure 1. Figure 2b illustrates a series of key frames from a movie depicting the automated library preparation workflow for a sample containing human gDNA from peripheral blood mononuclear cells (see video S1 for the complete execution of the protocol from end-to-end). First, the tagmentation reaction is performed by mixing the sample droplet and Nextera enzyme mix, aspirating, and incubating in a thermal module at 55°C for 5 minutes. A magnetic bead cleanup is subsequently performed by mixing the reaction product with magnetic beads. Using the magnetic separation module described above, the supernatant (containing excess enzyme and small DNA fragments) is removed, and the purified “tagmented” DNA is eluted from the beads and returned to the DMF device. Next, the library is mixed with PCR reaction mix, and limited-cycle PCR is performed in the thermal cycling module, to specifically amplify and enrich properly tagged fragments and incorporate Illumina-specific adapters at their ends. Finally, a two-stage size selection and clean-up process is performed using SPRI beads and the magnetic separation module, with bead binding conditions set to remove small DNA fragments (<200 bp) and then large DNA fragments (>400 bp). The resulting DNA library has a size distribution of 200–400 bp, ideal for Illumina sequencing.

**Figure 4:**
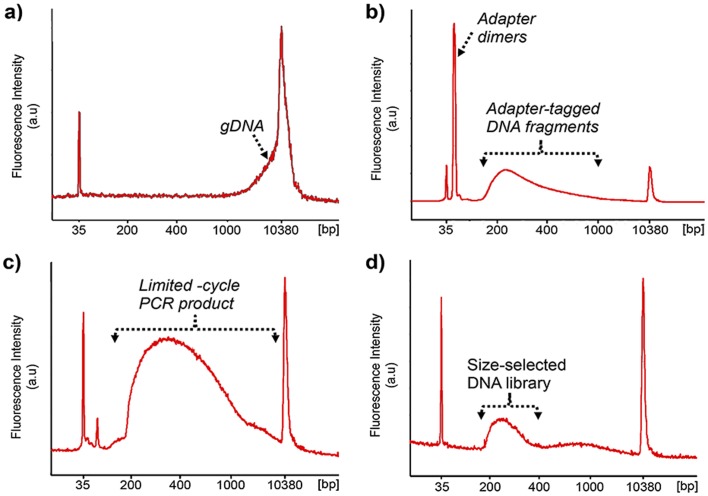
Analysis of human gDNA at different stages of the Nextera protocol. Bioanalyzer traces of a) gDNA, b) post-tagmentation, c) post-limited-cycle PCR and d) post-size-selection. Peaks at 35 and 10380 bp represent low- and high-molecular weight markers.

To qualitatively evaluate the effectiveness of our system for preparing sequencer-ready human gDNA libraries, we used a Bioanalyzer instrument (Agilent) to assess the size distribution of libraries generated from human gDNA at different stages of the Nextera protocol. The Bioanalyzer trace of the input gDNA sample (Figure 4a) shows a prominent peak in the high molecular weight region (>10,000 bp, overlapping with the 10,380 bp size standard), as expected for unfragmented gDNA. After tagmentation (Figure 4b), the majority of reaction products fell within a broad band of 150–1000 bp, with little high molecular weight DNA remaining. An additional peak at ∼45 bp corresponds to adapter dimers, which are routinely observed post-tagmentation [42] and readily removed by a subsequent clean-up step (trace not shown). Following limited-cycle PCR (Figure 4c), a broad band in the ∼ 200–10,000 bp region corresponds to the size distribution of enriched DNA fragments tagged with sequencing primers and indices. An additional peak at ∼55 bp corresponds to primer dimers that formed during PCR [42]. Finally, two-step size selection recovered adapter-bearing fragments that mainly fell within the desired 200–400 bp size range, though a small, broad hump in the baseline indicates that some higher molecular weight or single stranded products remained (Figure 4d). As shown, this method is a qualitative success, transforming a human gDNA sample into a properly sized sequencer-compatible library.

While our device produces very uniform libraries, there are many circumstances downstream of the device that can lead to variability in the number and quality of reads generated on the sequencer. The primary source of variation is the quantitation of the library and subsequent dilution and loading of the library into the sequencer. Because the first step after quantitation involves a ∼1000X dilution, even very small deviations in starting concentration can lead to large fluctuations in cluster density on the flow cell. Over-clustering tends to produce poor quality reads, while under-clustering will produce significantly fewer high-quality reads.

The new system reported here has several advantages relative to conventional techniques. An obvious advantage is automation and potential for high-throughput – a single technologist using our system could potentially do the work of several technologists using manual techniques. Furthermore, for the applications demonstrated here (for preparing a sequencing-ready library), the new system facilitate a reduction in amount of DNA sample (5 ng vs. 50 ng [43]–[45]), reagent use (∼ 30 µL vs. 80–650 µL [21], [44]) and analysis time of ∼ 1 h (5 min of hands-on time) vs. 1.5 h (15 min of hands-on time) [21], [43]. Our platform automates sample handling steps in the low microliter regime, which is difficult to perform reliably using manual or robotic pipetting. This capability is important because we can maintain the same optimized stoichiometry as bench scale handling (*i.e.* utilize the same ratio of input DNA to Nextera mix, same amount of beads, *etc*.), but at a volume scale where conventional pipettes and microcentrifuge tubes would introduce scale-dependent difficulties including evaporation, irreproducible delivery of reagents, and difficulties in visualizing or washing a pellet of magnetic beads. We have previously demonstrated the potential for performing capillary electrophoretic separation of sequencing libraries directly through one of the in-plane capillaries for providing online quality control monitoring of the library preparation process [20]. Although we have not included separation functionality in the current demonstration, its implementation as part of an automated workflow would further streamline the library preparation process by eliminating the need for bench-top characterization steps with stand-alone analytical instruments like the Bioanalyzer.

We also applied our automated library preparation technique to bacterial gDNA, derived from the well-characterized *Escherichia coli* strain MG1655 as well as *Klebsiella pneumoniae* strain BAA-2146, a multi-drug resistant clinical isolate of unknown genomic sequence. Bioanalyzer traces for final libraries prepared from these bacterial gDNA samples are shown in Figure S3. As with the human gDNA libraries, the bacterial gDNA libraries showed the desired 200–400 bp size distribution.

### Next Generation Sequencing Analysis

**Figure 5:**
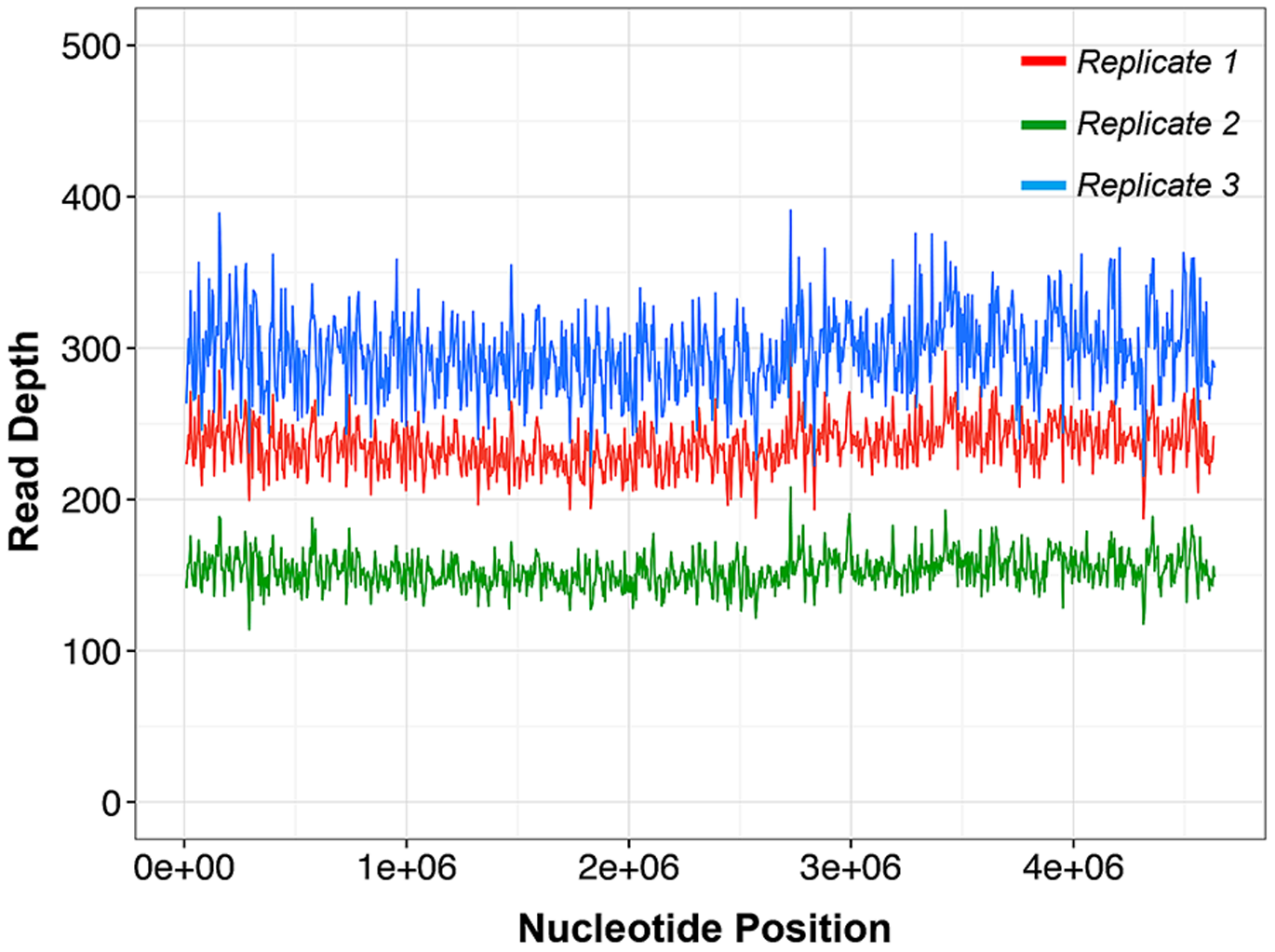
Genome coverage plots for three E. coli libraries prepared by microfluidic method. The plot represents relative depth of sequence coverage across the entire ∼4.6 kb *E. coli* genome.

The bacterial gDNA libraries were sequenced using a MiSeq (Illumina) instrument, which has throughput appropriate for complete sequencing of a bacterial genome in a single run. As shown in Table 1, the genome assembly metrics for bacterial whole-genome libraries prepared using our microfluidic method resulted in sequence data with greater than 99% of the reads mapping to the bacterial genome, leading to coverage depths of ∼168X over the entire genome. In addition, the coverage of the genome appeared to be very even, without any significant spikes or drops in coverage depth (Figure 5). De *novo* genome assembly yielded averages over three replicate runs of 249±27 for contig count and 62.1±18.2 kbp for N50 (a “median” contig length, defined as the contig length for which 50% of bases in the contig set are part of contigs larger than N50) as shown in Table S1. The quantitative metrics for *E. coli* strain MG1655 demonstrates that our microfluidic sample preparation technique yields high-quality sequencing data comparable to benchtop methods [46], [47] with only a fraction of the sample required by conventional methods.

**Table 1:** Sequencing metrics (pair-end 150 bp) of *E. coli* (strain MG1655) libraries prepared by microfluidic method aligned to reference genome.

Metrics	Replicate 1	Replicate 2	Replicate 3
Total Bases (GB)	1.1	0.7	1.3
Total Reads (M)	9.7	6.5	10.8
Mean Coverage* (X)	249	168	320
Reads Mapped to Reference (%)	99.4	99.2	99.7
% GC Content	50.1	49.7	50.1

* Mean coverage indicates the average depth of coverage at any given position within the *E. coli* genome.

In addition to sequencing a well-characterized *E. coli* strain, we tested our platform for a “real-world” application increasingly common in clinical microbiology: *de novo* sequencing of a novel bacterial strain. We selected the previously unsequenced *Klebsiella pneumoniae* ATCC BAA-2146 (KpnNDM), the first U.S. *Klebsiella* isolate found positive for the New Delhi metallo-beta-lactamase (NDM-1). De novo assembly of this genome was poorer than for the *E. coli* tests, yielding 1384 contigs with N50 of 12.4 kbp. This may be attributed in part to the quality of the purchased gDNA, the quality of this particular MiSeq run, or the intrinsic properties of the KpnNDM genome. These latter include its greater expected length (∼5.8 Mbp) than *E. coli* (4.6 Mbp), its expected higher GC content (∼57%) than *E. coli* (50.8%) which can affect Illumina sequencing, and its more numerous repeats that are long enough to block assembly. Nonetheless, the assembly provides evidence of multiple plasmids in the KpnNDM genome, including a small, high-copy number plasmid that we fully assembled, and a large number of transposable elements. *De novo* analysis also revealed at least five β-lactamase genes including NDM-1 which confers resistance to carbapenems, the “drugs of last resort” for Gram-negative bacterial infections. Data for the KpnNDM genome assembly is available at National Center for Biotechnology Information (accession number APNN00000000.1). Detailed analysis of the KpnNDM genome, incorporating additional technologies to close gaps in the assembly, will be the subject of a separate manuscript.

### Conclusions

We introduce here a fully integrated and automated microfluidic system, in which the DMF platform serves as a “hub” to integrate the operation of off-DMF modules for preparing DNA libraries for personal sequencers. To validate the capabilities of the new method, DNA libraries were prepared by the new method and sequences of libraries were generated using an in-house sequencer. The integrated microfluidic method has the potential to become a powerful new tool to enable next generation sequencing studies in many laboratories, particularly in smaller laboratories where high-throughput robotic liquid handling stations are not appropriate. Key applications include analysis of clinical specimens or environmental samples, which are inherently unique and limited in quantity, and thus require precision handling at small scale.

## Supporting Information

Figure S1 **Effect of Pluronic F127 (in Nextera enzyme solution) on tagmentation reaction.** An gDNA-Nextera enzyme reaction was allowed to proceed at 55°C for 5 min in a microcentrifuge tube under two conditions: a) without and b) with Pluronic added to the Nextera enzyme solution. The bioanalyzer traces for the two products are comparable, which indicates that the activity of the enzyme is unaffected by the addition Pluronic.
(TIF)

Figure S2 **Temperature traces of processing module and thermal block cycler over time.**
(TIF)

Figure S3 **Bioanalyzer trace of sequencer-ready a)** ***Escherichia coli*** **and b)** ***Klebsiella pneumoniae*** **gDNA library.** Peaks at 35 and 10380 bp represent low- and high-molecular weight markers.
(TIF)

Table S1 ***De novo*** **sequencing and assembly metrics of** ***E. coli*** **MG1655 and** ***K. pneumoniae*** **ATCC BAA-2146 libraries prepared by the microfluidic method.**
(DOCX)

Video S1 **This video depicts the automated library preparation workflow for a sample containing human gDNA.**
(WMV)
