# Supplementary figures and images for: A Microfluidic DNA Library Preparation Platform for Next-Generation Sequencing
Source: PLoS One. 2013 Jul 22;8(7):e68988. doi: 10.1371/journal.pone.0068988 (PMC3718812; doi:10.1371/journal.pone.0068988)

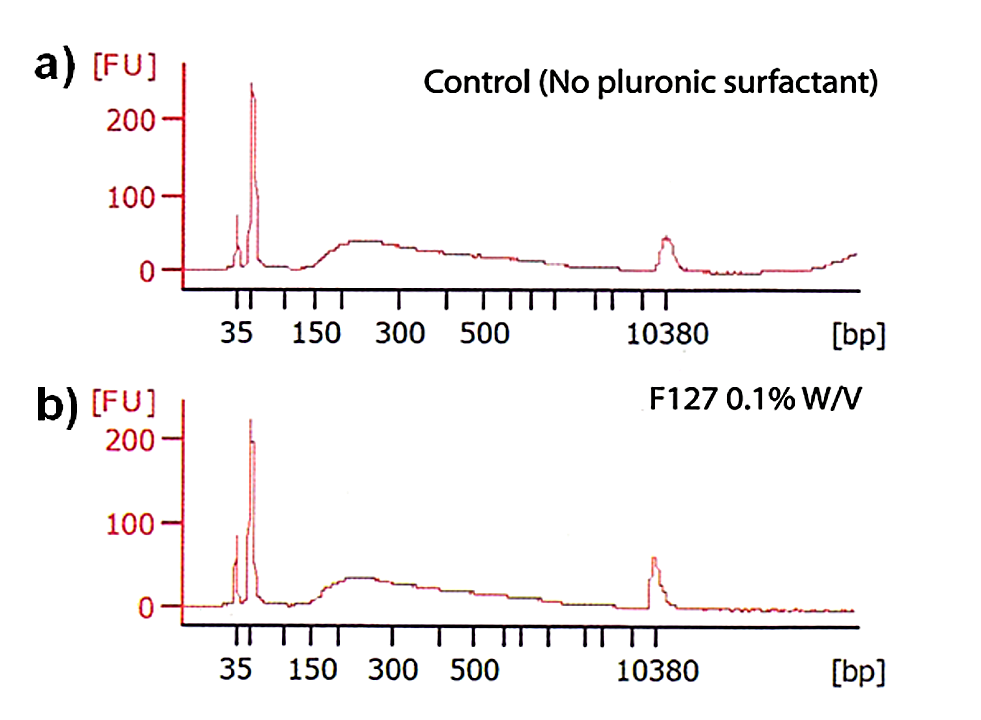

Supplement: Figure S1 — Effect of Pluronic F127 (in Nextera enzyme solution) on tagmentation reaction. An gDNA-Nextera enzyme reaction was allowed to proceed at 55°C for 5 min in a microcentrifuge tube under two conditions: a) without and b) with Pluronic added to the Nextera enzyme solution. The bioanalyzer traces for the two products are comparable, which indicates that the activity of the enzyme is unaffected by the addition Pluronic. (TIF) [file pone.0068988.s001.tif]

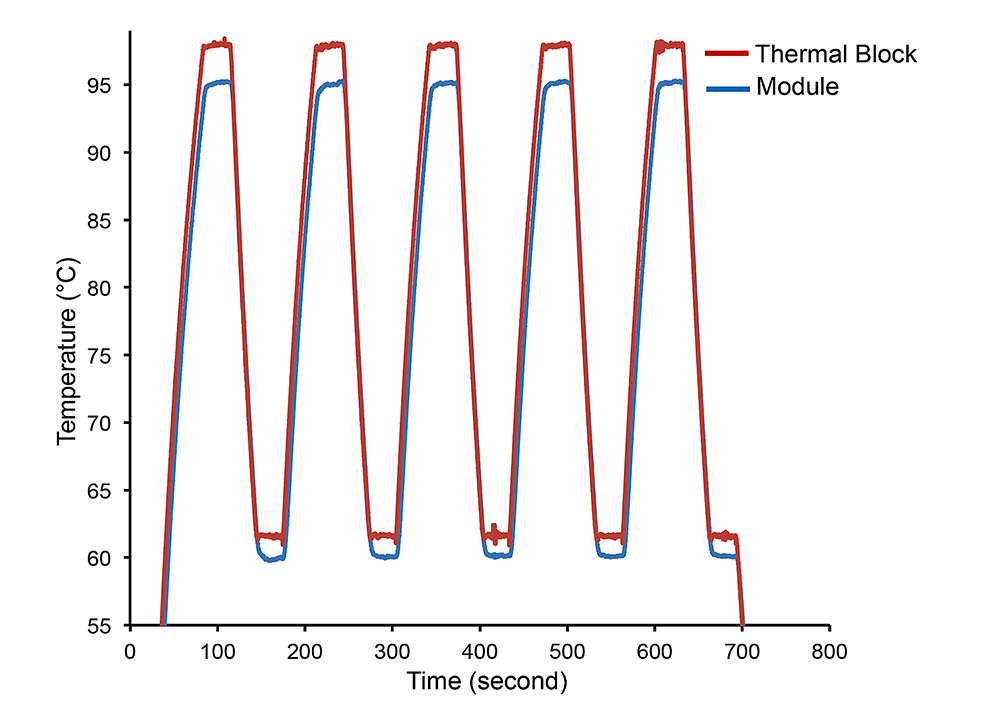

Supplement: Figure S2 — Temperature traces of processing module and thermal block cycler over time. (TIF) [file pone.0068988.s002.tif]

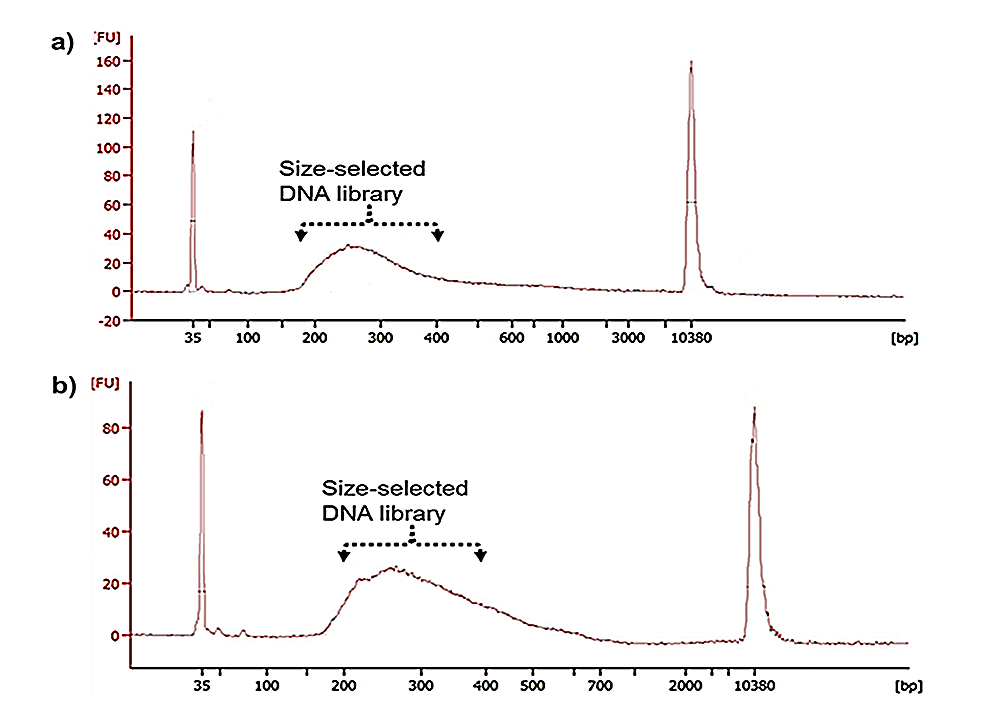

Supplement: Figure S3 — Bioanalyzer trace of sequencer-ready a) Escherichia coli and b) Klebsiella pneumoniae gDNA library. Peaks at 35 and 10380 bp represent low- and high-molecular weight markers. (TIF) [file pone.0068988.s003.tif]
