# Supplementary material for: A Microfluidic DNA Library Preparation Platform for Next-Generation Sequencing
Source: PLoS One. 2013 Jul 22;8(7):e68988. doi: 10.1371/journal.pone.0068988 (PMC3718812; doi:10.1371/journal.pone.0068988)
Supplement: Table S1 — De novo sequencing and assembly metrics of E. coli MG1655 and K. pneumoniae ATCC BAA-2146 libraries prepared by the microfluidic method. (DOCX) [file pone.0068988.s004.docx]

|  | ***E. coli* 1** | ***E. coli* 2** | ***E. coli* 3** | ***K. pneum*.** |
| --- | --- | --- | --- | --- |
| **Quality Filter** |  |  |  |  |
| No. Raw Read Pairs | 5582300 | 3771505 | 7008619 | 3308598 |
| Rejected (%) | 9.2 | 8.4 | 16.2 | 8.6 |
| **De Novo Assembly Inputs** |  |  |  |  |
| No. Read Pairs | 5067679 | 3453625 | 5869837 | 3023757 |
| Mean Read Length (bp) | 113.6 | 109.0 | 123.7 | 88.3 |
| Mean Insert Length (bp) | 190 | 189 | 193 | 197 |
| Exp. Genome Length (kbp) | 4640 | 4640 | 4640 | 5799 |
| Exp. Coverage | 248.0 | 162.2 | 312.8 | 92.0 |
| k | 25 | 29 | 29 | 29 |
| **Contigs > 200 bp** |  |  |  |  |
| No. | 233 | 280 | 233 | 1384 |
| Mean Length (kbp) | 19.4 | 16.1 | 19.4 | 4.1 |
| N50 (kbp) | 57.2 | 46.8 | 82.3 | 12.4 |
| Total Length (kbp) | 4509 | 4503 | 4525 | 5611 |
